# Marital status and long-term cardiovascular risk in general population (Gubbio, Italy)

**DOI:** 10.1038/s41598-023-33943-0

**Published:** 2023-04-25

**Authors:** Xavier Humbert, Andry Rabiaza, Sophie Fedrizzi, Joachim Alexandre, Alessandro Menotti, Emmanuel Touzé, Martino Laurenzi, Oscar Terradura-Vagnarelli, Paolo E. Puddu

**Affiliations:** 1 Département de medecine generale, Normandie Université, UNICAEN, 14000 Caen, France; 2 INSERM U1086 ANTICIPE, Normandie Université, UNICAEN, 14000 Caen, France; 3 EA 4650, Signalisation, électrophysiologie et imagerie des lesions d’ischémie reperfusion myocardique, UNICAEN, 14000 Caen, France; 4 Service de Pharmacologie, Normandie Université, UNICAEN, CHU Caen Normandie, 14000 Caen, France; 5 Association for Cardiac Research, 00198 Rome, Italy; 6 Service de Neurologie, Normandie Université, UNICAEN, CHU Caen Normandie, 14000 Caen, France; 7 INSERM U1237 PhIND, Normandie Université, UNICAEN, 14000 Caen, France; 8 Centro Studi Epidemiologici di Gubbio (CeSEG), 06024 Gubbio, Perugia, Italy; 9 Département de medecine generale, Pôle de formation et de recherche en sante, 2, rue des Rochambelles, 14000 Caen, France

**Keywords:** Circulation, Cardiology

## Abstract

To investigate whether marital status is associated to long-term major fatal and non-fatal cardiovascular events in men and women from the Gubbio Population Study. The incidence of cardiovascular disease (CVD), including stroke and coronary heart disease (CHD) and CVD death together with all-cause mortality were analyzed. The analysis included 2832 persons (44% men, 54 ± 11 years old). Marital status was defined at entry as married (married or living conjugally) versus unmarried subjects (widowed, separated, divorced or single). Married and unmarried subjects did not differ concerning socio-demographic, anthropometric and biological variables at baseline. Over 191 months median follow-up, the incidence of CHD was lower among married versus unmarried women [HR: 0.63 (95% CI 0.41–0.96)] only; the same was true for CHD mortality [HR: 0.43 (95% CI 0.22–0.84)] and all-cause mortality [HR: 0.75 (95% CI 0.59–0.96)] independently of traditional risk factors (age, SBP, total and HDL cholesterol, cigarette smoke and BMI). In men, marital status was not associated to any of the investigated outcomes. In primary care, marital status should be investigated as it can be associated with long-term CHD and all-cause incidence and mortality risks among women.

## Introduction

Substantial progress has been made in understanding the epidemiology, pathophysiology, and risk associated with cardiovascular disease (CVD). CVD is associated with significant morbidity and mortality^1^. In this context, traditional cardiovascular (CV) risk factors including age, sex, hypertension, hyperlipidemia, smoking and diabetes mellitus have been previously identified; these risk factors together explain 80% of the risk of developing CVD^2^. However, the determinants for the remaining 20% risk remain unclear^2,3^.

Influence of socio-economic risk factors on risk of CVD morbidity and mortality may have a role, in particular marital status^4^. The benefits of marital status on health and mortality have been inconsistently demonstrated^5,6^. In some studies of the general population, married subjects seem to have a better prognosis after myocardial infarction^5,7–11^ and stroke^12,13^, whereas other studies demonstrated no influence of marital status^14–16^. Moreover, marital status has generally been demonstrated more protective in men than in women^17,18^. However, this link has been poorly demonstrated in populations with a long-term follow-up (more than 10 years)^4,19,20^.

The aim of this analysis was to investigate whether in the Gubbio Population Study marital status is associated to long-term major non-fatal and fatal CVD and mortality in men and women.

## Methods

### Population

For this investigation, we have used data extracted from the Gubbio Population Study. Their methods and main results have been previously reported^21^. In brief, Gubbio is an ancient hill town in central Italy, at the lower reaches of the Apennine mountain chain. This Umbrian town has a well-preserved wall-enclosed central area. An invitation to participate in the study was extended to the entire population aged 5 years and over residing within the walls. Names and addresses were provided by the municipal authority through the census lists. Following an invitation letter signed by the town’s mayor, explaining the nature of the study, an employee visited each family to illustrate the study’s aims and procedures and to make an appointment for all family members willing to participate. In order to gather data into families, the invitation was extended to first-degree relatives (parents, siblings and offspring) residing outside the medieval city walls. A baseline examination (Exam 1) was performed between 1983 and 1986, with the participation of a total of 5376 individuals with a response rate exceeding 93%. The first follow-up examination (Exam 2) was performed between 1989 and 1992. The second follow-up examination (Exam 3) was performed between 2001 and 2007. Only individuals aged 35 through 74 years at Exam 1 were considered for this analysis, for a total of 3124 individuals.

### Baseline examination

A comprehensive baseline medical examination was performed including a battery of standard laboratory tests and measurements of special interest to the Gubbio Population Study in view of its focus on hypertension (HT)^21^. Verbal consent was obtained from participants in compliance with the Helsinki Declaration. A questionnaire on lifestyle and health problems was administered, and a number of anthropometric, biochemical, biophysical and medical measurements were made^21^. The following measurements and information collected at the baseline examination were used for the present analysis: age, sex, HT history, current use of antihypertensive drugs, systolic blood pressure (SBP) and diastolic blood pressure (DBP) measured according to the standard World Health Organization (WHO) protocol, total serum cholesterol, high-density lipoprotein cholesterol (HDL), smoking habits, history of diabetes mellitus; fasting blood glucose (FBG), body mass index (BMI), heart rate (HR), urine creatinine, and serum uric acid. Biochemical measurements were assayed by enzymatic methods that was measured by kinetic alkaline picrate assay: they were partly under external control of the WHO Lipid Reference Center of Prague.

### Marital status

At inclusion, marital status was recorded by questionnaire, consisting of 7 categories: married, conjugal living, single, widowed, separated, divorced or unknown. In the present analysis, we classified the population at entry into two categories: married subjects – which included married and living in conjugal status and unmarried – i.e. subjects who were single, widowed, separated or divorced. Subject with unknown status were excluded.

### Follow-up and outcome definition

The follow-up was conducted up to 20 years after the entry exam. CVD was identified using data collected in subsequent examinations and -for those who did not attend -it was identified with home visits, review of hospital records, interview with subjects, family physicians and/or telephone interview. Subjects were classified as lost to follow-up after systematic telephone search, home visits, and review of local municipality registers assigning a date when presumably last seen alive. Six end-points were considered for this analysis: (1) incidence of non-fatal and fatal CVD, including coronary heart disease (CHD), stroke, peripheral artery disease and heart disease of uncertain etiology (HDUE) including cases of heart failure, severe arrhythmias and blockades in the absence of a clear CHD with typical characteristics. Cases characterized only by angina pectoris, intermittent claudication and transient ischemic attacks were not classified in this group; (2) incidence of CHD, including non-fatal and fatal myocardial infarction and sudden coronary death (when other causes could be reasonably excluded); (3) non-fatal and fatal stroke (STR) for which it was not possible to differentiate ischemic versus hemorrhagic strokes; (4) CVD death, including CHD deaths, and fatal cases of stroke, peripheral artery disease and HDUE; (5) CHD death including fatal myocardial infarction and sudden coronary death; (6) all deaths.

In all cases, only the first CVD episode was used for the analysis and subjects with previous CVD disease were exluded. Deaths and major non-fatal CVD cases were coded following the 9th Revision of the WHO-International Classification of Diseases^22^. Details on the diagnostic procedures and criteria are reported elsewhere^23,24^.

### Statistical analysis

Data were expressed as means ± SD or percentages. Comparisons between groups were made using Student’s *t* test or the Chi^2^ test, as appropriate. Survival curves were estimated using the Kaplan–Meier product-limit method and were compared by the Mantel logrank test in all-death and CHD death analysis. The effect of selected covariates (age, sex, SBP, TC, HDL, cigarettes, BMI and marital status) on survival was evaluated by Cox Proportional Hazards model (Efron ties method) using NCSS version 9 (Hintze J, Kaysville, Utah, USA: www.ncss.com). Adjusted hazard ratios [HR ± 95% confidence intervals, (CI)] were calculated sex-wise by considering 6 covariates (age, BMI, total cholesterol, HDL, cigarettes smoked per day, and SBP) selected on the basis of previous works^23–27^.

### Ethics approval and consent to participate

The Authors declare that all procedures were carried out following standard protocols and in accordance with all relevant guidelines and regulations. The Gubbio Population Study adheres to the Declaration of Helsinki. Ethical approval was given by the Ethical Committee of the Local Health Authority of Alto Chiascio (Perugia, Italy) which was followed by the Ethical Committee of the Regional Authority of Umbria (reference #2850/16). Informed written consent was given by and filed from all participants.

## Results

**Figure 1:**
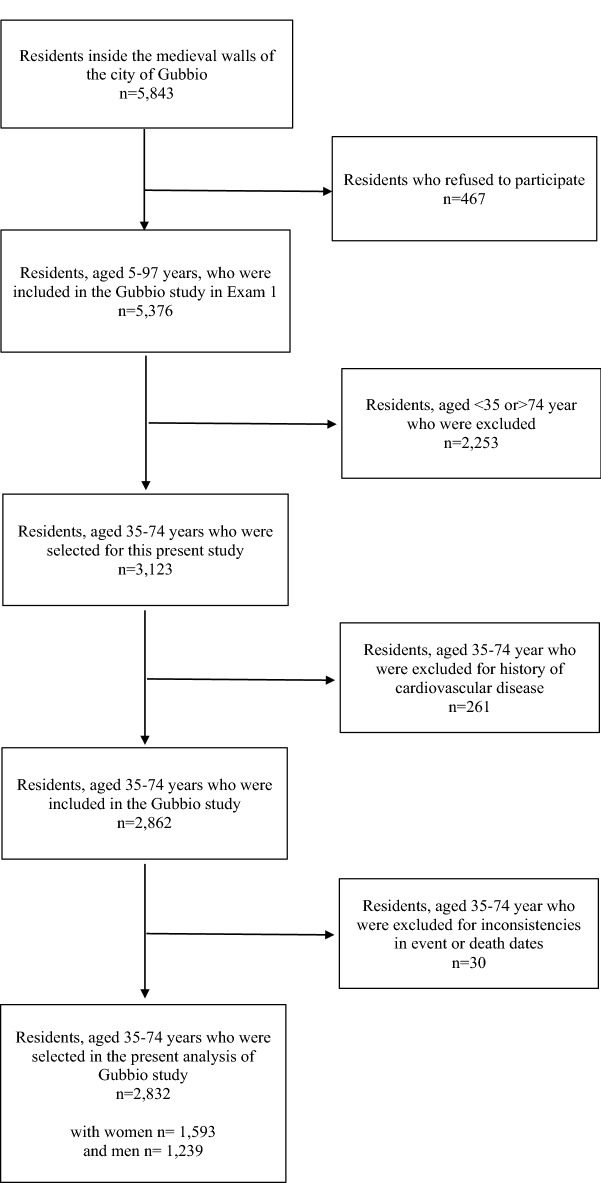
Flow chart of the present analysis of the Gubbio Population Study.

Among the 5376 subjects aged 5–97 years who participated to the Gubbio Population Study (Exam 1) between 06/01/1983 and 20/12/1985, the 3,123 aged 35–74 years were selected here whereas 261 subjects were excluded for CVD history and 30 subjects were also excluded for inconsistencies in event or death dates or missing data. Consequently, 2832 individuals were included in analyses (1593 women and 1239 men) (Fig. 1). The median follow-up was of 15.9 years (interquartile range 12 to 19 years), roughly corresponding to > 50,000 person-years. 481 CV events were observed including 228 coronary heart events (47.4%) and 180 STRs (37.4%). In term of mortality, 555 deaths were observed including 220 CV deaths (39.6%) [84 CHD deaths (38.1% of CVD deaths) and 77 STR deaths (35.0% of CVD deaths)].

There were 1183 unmarried subjects with 932 single subjects, 243 widowed subject and 8 divorced/separated subjects. On the other hand, there were 1649 married subjects. Table 1 shows that there was no significant difference in measured risk factors between married (n = 1649, 58.2%) and unmarried individuals (n = 1183, 41.8%), at baseline. Conversely, married women were more frequently diabetic (p = 0.03) while FBG was similar in married and unmarried women (p = 0.73) at inclusion.

**Table 1:** Baseline values by sex and marital status in the participants in the Gubbio Population Study.

	Women			Men			All		
	Married	Unmarried	p-value	Married	Unmarried	p-value	Married	Unmarried	p-value
Sex (M/F, %)	NA	NA	NA	NA	NA	NA	932/717 (0.43)	661/522 (0.44)	0.73
Age (years)	54 ± 11	55 ± 11	0.19	53 ± 11	53 ± 11	0.80	54 ± 11	54 ± 11	0.30
Actual status of HBP, no. (proportion)	427/932 (0.46)	197/661 (0.30)	0.67	292/717 (0.41)	206/522 (0.40)	0.7	719/1649 (0.44)	516/1183 (0.44)	0.99
Treated hypertensive subjects	265/932 (0.28)	310/661 (0.47)	0.55	142/717 (0.20)	103/522 (0.20)	0.97	407/1649 (0.25)	300/1183 (0.25)	0.71
SBP (mmHg)	139 ± 24	138 ± 24	0.86	135 ± 21	135 ± 21	0.74	137 ± 23	139 ± 23	0.70
DBP (mmHg)	80 ± 11	80 ± 12	0.78	81 ± 11	80 ± 10	0.41	80 ± 11	80 ± 11	0.80
Total cholesterol (mg/dl)	221 ± 43	221 ± 42	0.90	218 ± 44	219 ± 39	0.64	220 ± 43	220 ± 41	0.83
HDL (mg/dl)	51 ± 12	51 ± 12	0.78	43 ± 11	43 ± 11	0.37	48 ± 12	47 ± 12	0.54
Cigarettes (n. per day)^**ª**^	2.4 ± 5	2.5 ± 5	0.63	8 ± 11	8 ± 11	0.63	4.7 ± 9	4.9 ± 9	0.98
Diabetes Y/N, no. (proportion)	85/906 (0.09)	39/623 (0.06)	0.03	45/689 (0.07)	39/505 (0.08)	0.49	130/1595 (0.08)	78/1128 (0.07)	0.26
FBG (mg/dl)	91 ± 20	91 ± 25	0.73	91 ± 19	93 ± 26	0.76	91 ± 19	92 ± 25	0.97
BMI (kg/m^2^)	27 ± 4	27 ± 5	0.96	27 ± 3	27 ± 4	0.86	27 ± 4	27 ± 4	0.84
HR (beats/min)	73 ± 10	74 ± 11	0.53	71 ± 11	70 ± 11	0.74	72 ± 11	72 ± 11	0.82
Urine creatinine (mmol/l)	111 ± 47	109 ± 45	0.32	150 ± 56	147 ± 53	0.16	128 ± 54	125 ± 52	0.18
Uric acid (mg/dl)	4.34 ± 1.33	4.43 ± 1.26	0.13	6 ± 1	6 ± 1	0.13	4.94 ± 1.57	5.04 ± 1.53	0.11

*BMI* body mass index, *DBP* diastolic blood pressure, *FBG* fasting blood glucose, *HDL* high-density lipoprotein, *HBP* high blood pressure, *HR* heart rate, *NA* non applicable, *NS* non-significant, *SBP* systolic blood pressure.

ªMedian value was 0.0 for both married and unmarried.

**Figure 2:**
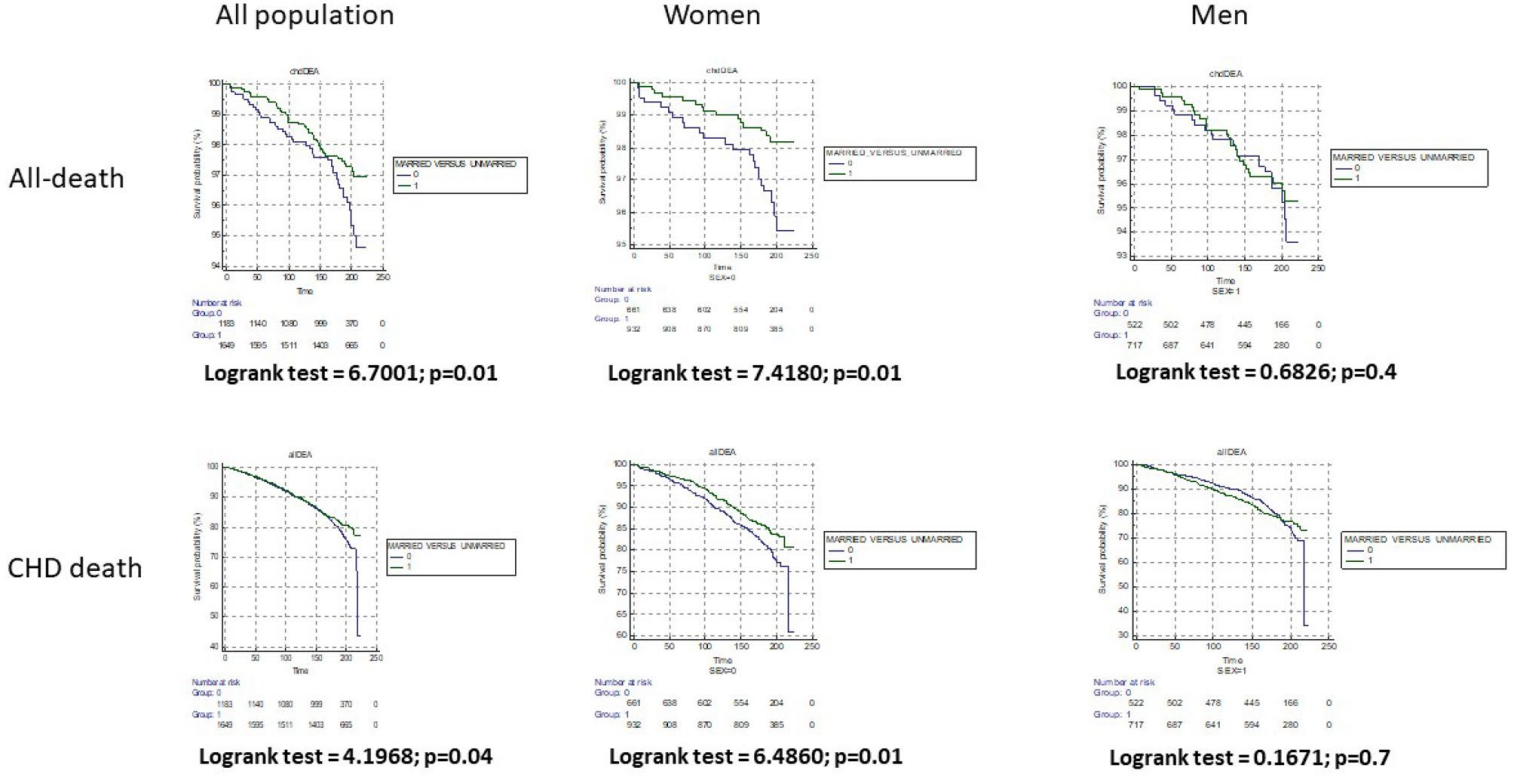
Kaplan–Meier graphs of crude all-death and coronary heart disease death rates during approximately 20 year in men and women from Gubbio Population Study in function of marital status (married versus unmarried). *CHD* coronary heart disease.

Figure 2 illustrates the Kaplan–Meier graphs of crude outcomes during approximately 20 years as a function of marital status: significant logrank tests are seen for CHD (logrank test = 4.1968, p = 0.04) and all-cause death (logrank test = 6.7001, p = 0.01) in the total population. Similar results were observed in women but not in men. It is notable that no difference exists in the all death Kaplan Meier curve (all-population) until 160–170 months of follow-up. Moreover, we have noted a risk crossing in men after 180 months of follow-up in the all-death Kaplan Meier curve.

**Figure 3:**
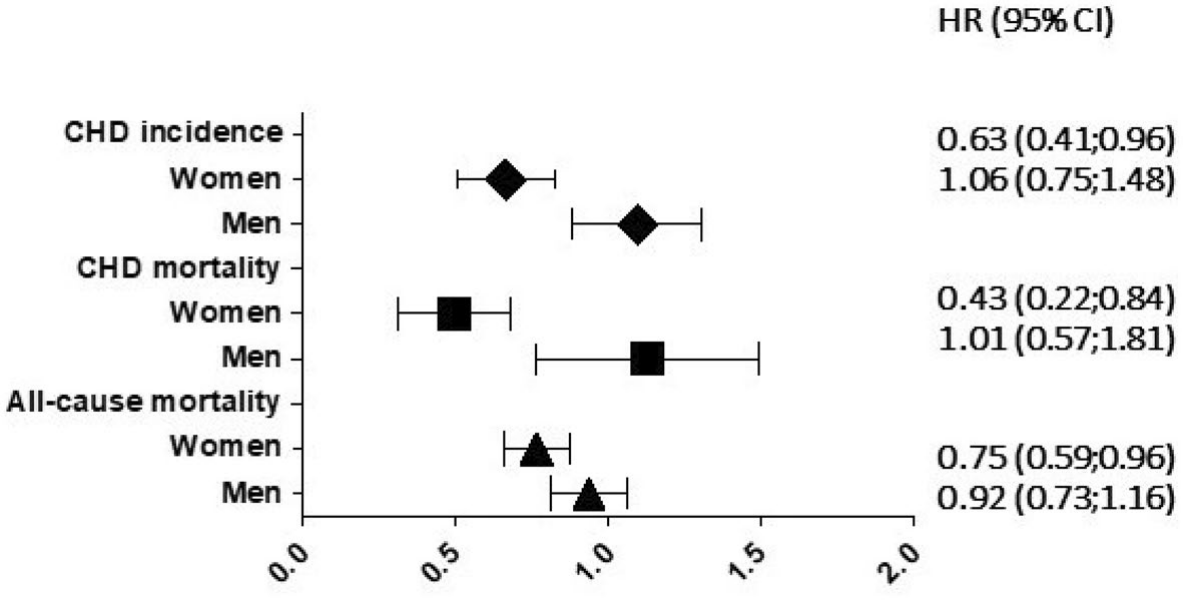
Marital status hazard ratios for three out of seven outcomes in approximately 20 years in the Gubbio Population Study, adjusted for age, SBP, total and high-density lipoprotein cholesterol, cigarettes and BMI in women and in men. See also Tables 2 and 3.

Table 2 presents the hazard ratio and 95% CI of Cox’s model solution in women; it clearly shows that in presence of traditional CV risk factors, married status is positively associated to the incidence of CHD [HR 0.63 (0.41;0.96)], and of CHD- [HR 0.43 (0.22;0.84)] and all-causes mortality [HR: 0.75 (0.59;0.96)]. In men, marital status was not associated to any of the 6 outcomes (Table 3 and Fig. 3).

**Table 2:** Hazard ratios (HR) and 95% confidence intervals (CI) of cox’s model solutions (Efron’s ties method), computed on units of covariates, to study association of many factors with incident events (fatal and non-fatal) or death in all-women of the Gubbio Population Study during 191 months of median follow-up.

Covariables	Mean	Incidence of			Death due to			
		CVD	CHD	STR	CVD	CHD	STR	All-cause
		HR (95% CI)	HR (95% CI)	HR (95% CI)	HR (95% CI)	HR (95% CI)	HR (95% CI)	HR (95% CI)
Age (years)	54.80	1.11 (1.09; 1.14)	1.08 (1.05; 1.11)	1.12 (1.09; 1.16)	1.16 (1.13; 1.20)	1.10 (1.05; 1.15)	1.20 (1.14; 1.27)	1.12 (1.10; 1.14)
SBP (mmHg)	138.63	1.01 (1.00; 1.02)	1.00 (0.99; 1.01)	1.01 (1.00; 1.02)	1.01 (1.00; 1.02)	1.01 (0.99; 1.03)	1.00 (0.99; 1.02)	1.01 (1.00; 1.02)
TC (mg/dL)	221.15	1.00 (0.99; 1.01)	1.00 (1.00; 1.01)	1.00 (0.99; 1.01)	1.00 (0.99; 1.01)	1.01 (1.00; 1.02)	0.99 (0.98; 1.00)	0.99 (0.98; 1.00)
HDL (mg/dL)	50.67	0.98 (0.97; 0.99)	0.97 (0.95; 0.99)	0.99 (0.97; 1.01)	0.98 (0.96; 0.99)	0.97 (0.94; 0.99)	0.99 (0.96; 1.02)	0.99 (0.98; 1.00)
Cigarettes (N/day)	2.42	1.02 (0.99; 1,06)	1.04 (1.00; 1.08)	1.01 (0.96; 1.06)	1.05 (1.01; 1.10)	1.05 (0.99; 1.11)	0.93 (0.79; 1.09)	1.05 (1.02; 1.07)
BMI (kg/m^2^)	27.46	1.00 (0.97; 1.03)	1.00 (0.95; 1.05)	0.99 (0.94; 1.03)	0.98 (0.94; 1.02)	0.94 (0.86; 1.01)	0.96 (0.90; 1.03)	0.98 (0.95; 1.01)
Marital status (married: 1; other: 0)	0.58	0.77 (0.59; 1.00)	0.63 (0.41; 0.96)	0.80 (0.53; 1.19)	0.76 (0.52; 1.11)	0.43 (0.22; 0.84)	1.10 (0.59; 2.05)	0.75 (0.59; 0.96)
Processed		1528	1528	1528	1528	1528	1528	1528
Failed		216	87	97	108	36	43	260
Censored		1312	1441	1431	1420	1492	1485	1268
Log likelihood		− 1404.18	− 577.18	− 623.94	− 667.18	− 229.58	− 253.10	− 1700.72

*SBP* systolic blood pressure (the average between the second and third measurement), *TC* total cholesterol, *HDL* high-density lipoprotein cholesterol, *BMI* body mass index, *CVD* cardiovascular disease by hard criteria, *CHD* coronary heart disease by hard criteria, *HR* hazard ratio, *STR* stroke.

**Table 3:** Hazard ratios (HR) and 95% confidence intervals (CI) of cox’s model solutions (Efron’s ties method), computed on units of covariates, to study association of many factors with incident events (fatal and non-fatal) or deaths in all-men of the Gubbio Population Study during 191 months of median follow-up.

Covariables	Mean	Incidence of			Death due to			
		CVD	CHD	STR	CVD	CHD	STR	All-cause
		HR (95% CI)	HR (95% CI)	HR (95% CI)	HR (95% CI)	HR (95% CI)	HR (95% CI)	HR (95% CI)
Age (years)	53.06	1.06 (1.05; 1.08)	1.02 (1.00; 1.04)	1.12 (1.09; 1.15)	1.12 (1.09; 1.15)	1.06 (1.02; 1.09)	1.20 (1.13; 1.28)	1.12 (1.10; 1.13)
SBP (mmHg)	134.91	1.02 (1.01; 1.03)	1.02 (1.01; 1.03)	1.01 (1.00; 1.02)	1.02 (1.01; 1.03)	1.03 (1.01; 1.04)	1.01 (0.99; 1.03)	1.01 (1.00; 1.02)
TC (mg/dL)	218.51	1.01 (1.00; 1.02)	1.01 (1.00; 1.02)	1.00 (0.99; 1.01)	1.00 (0.99; 1.01)	1.01 (1.00; 1.02)	1.00 (0.98; 1.01)	0.99 (0.98; 1.00)
HDL (mg/dL)	43.36	0.99 (0.98; 1.00)	0.99 (0.97; 1.01)	1.01 (0.99; 1.03)	0.99 (0.97; 1.01)	0.98 (0.95; 1.01)	1.02 (0.99; 1.05)	1.00 (0.99; 1.01)
Cigarettes (N/day)	7.85	1.02 (1.01; 1,04)	1.03 (1.01; 1.04)	1.02 (0.99; 1.04)	1.03 (1.01; 1.05)	1.04 (1.02; 1.06)	1.02 (0.97; 1.07)	1.03 (1.02; 1.04)
BMI (kg/m^2^)	27.12	1.04 (1.01; 1.08)	1.05 (1.01; 1.10)	1.05 (0.99; 1.12)	1.05 (1.00; 1.11)	1.06 (0.99; 1.14)	1.03 (0.94; 1.14)	1.00 (0.98; 1.04)
Marital status (married: 1; other: 0)	0.58	1.11 (0.87; 1.43)	1.06 (0.75; 1.48)	1.08 (0.69; 1.68)	1.10 (0.75; 1.61)	1.01 (0.57; 1.81)	1.27 (0.62; 2.58)	0.92 (0.73; 1.16)
Processed		1218	1218	1218	1218	1218	1218	1218
Failed		265	141	83	112	48	34	295
Censored		953	1077	1135	1106	1170	1184	923
Log likelihood		− 1711.44	− 931.94	− 504.41	− 671.18	− 298.49	− 182.26	− 1836.19

*SBP* systolic blood pressure (the average between the second and third measurement), *TC* total cholesterol, *HDL* high-density lipoprotein cholesterol, *BMI* body mass index, *CVD* cardiovascular diseases by hard criteria, *CHD* coronary heart disease by hard criteria, *STR* stroke.

## Discussion

Our analysis shows that in Gubbio Population Study at baseline there was no difference in CV risk factors between married versus unmarried subjects of both sex (Table 1). When the marital status was forced into a multivariate Cox’s model where standard risk factors were considered, there was a decreased 20-year CHD incidence risks by 37% and CHD and all-causes mortalities risks lower by 25% to 57%, respectively in married women only (Table 2 and Fig. 3).

The correlation of marital status with the risk of CVD has been previously investigated. A systematic review and metanalysis made by Wong et al. in 2018 comprised 34 studies published since 2000 that included more than two million people^1^. In their study, the risks of CVD [OR: 1.42 (1.00;2.01)], CHD [OR: 1.16 (1.04;1.28)] and CHD [OR: 1.43 (1.28;.60)] and STR [OR: 1.55 (1.16;2.08)] was higher for unmarried participants in both sex. The risk of incidence of CHD was higher among divorced participants although the risk of STR (p < 0.001) was increased among widowers. Mortality was higher [OR: 1.42 (1.14;1.76)] among single individuals with myocardial infarction.

Long-term follow-up residential cohorts have rarely been studied in regard to the relation between marital status and CVD. In our analysis, there was no difference in the all-death Kaplan–Meier curve at 12 years of follow-up. For the same follow-up duration, Akima et al. in an epidemiological study performed on 1609 subjects (795 men and 815 women, aged 25–64 at inclusion) showed a higher relative risk of CVD death among married women [RR: 3.21 (1.28;8.06), p < 0.001] and unmarried men [RR: 5.47 (1.69;17.74), p < 0.001]^4^. In a 18-year prospective cohort composed of 15,827 subjects (7,264 men and 8,563 women), Dupre et al. found a higher risk of CHD in divorced women [RR: 1.77 (1.30;2.41)] than in re-married women [RR: 1.35 (1.07;1.70)] after adjusting for multiple risk factors (age, study, race, ethnicity, geographic region, ever widowed, BMI, hypertension, diabetes, education, employment, income, health insurance, living alone, number of children, depression symptoms, smoking status, alcohol use and physical exercise). In this study, the risk of CHD was higher for divorced men [RR: 1.30 (1.02;1.66)] than for married ones^19^. Consequently, two major factors appear to influence the incidence of CVD and mortality which are linked to marital status: follow-up delay and determined marital status. The health status of individuals living alone was studied in different countries with different ways of life and culture^11^. Loneliness has been correlated to low social support and higher risk of social isolation^28^. People living alone have an increased risk of leading an unhealthy lifestyle and to not follow medical recommendations as they do not benefit of the care and financial help from others^29,30^. Their adherence to medical treatment is also undermined. On the psychological side, loneliness increases the risk of depression. Furthermore, a quicker access to care leading to a potentially better CV prognosis could be reached by the presence of a life partner^31^. The above explanations can be suggested to understand the poor health outcome among unmarried women in our study.

On the other side, for married people, marital stress can have a potential major negative impact on the prognosis of CVD. Women with a high marital stress score had a threefold higher risk of a new CHD event than ones without stress in a consecutive population based, prospective study of 292 women aged between 30 and 65 year-old hospitalized for acute myocardial infarction^11^. Orth-Gomer et al. suggested that women have a lower perceived support by their partner than men. Most frequent reported stressors were infidelity, alcohol abuse and partner’s illness. These were chronic, major and concrete^32^. Marital status is not the only source of stress. Work stress is also interesting even if its explanation is often affected by the focus on socially or economically privileged women. In addition, confusion can occur between both stresses as one third of the eligible women in Orth-Gomer study worked at home in the United States compared to almost none in Sweden^32^. Beyond the profession, domestic labor, including child and elderly care, are still mostly the prerogative of women in spite of their increasing participation in the workforce outside of the home^18^. Finally, health behaviors can be impacted by work and marital stress through the increased use of tobacco and alcohol and by negatively affecting dietary habits and sleep patterns^33,34^. However, in another study, the risk of falling into poverty and its adverse consequences was linked to marital breakup^35^.

Variation in methodology and study sample characteristics may partially explain the different findings of our analysis in comparison to previous investigations reported in the literature. Studies are conducted in different settings including hospital or community-based samples, and are often restricted to different groups of participants, such as employed or rehabilitated patients. Some studies are restricted to older patients while other are oriented to younger and healthier subjects. Some are designed as retrospective studies in which all data regarding medical conditions as well as psychosocial and other risk factors are extracted from medical records. We considered here the marital status only at inclusion and not during the follow-up, combining married and conjugal couples whereas the counterpart was formed by single, widowed, separated and divorced. A single assessment of marital status at baseline, instead of repeated ones during follow-up, however, is probably sufficient in our analysis of this long-term follow-up study because remarriage is a relatively rare event in the Mediterranean population especially during the 90’s (i.e. < 5%)^5^. It is known that among catholic and orthodox people marriage is strongly related to religion. Being married has been associated with financial stability, opportunities for social development, better dietary habits, and well-being^5^. While the definition of unmarried status is not always clear despite noted differences in the divorced or separate, widowed, and never-married groups in the literature, Schultz et al. previously showed that CV events are higher in each unmarried group (divorced/separate, widowed, and never married) versus the married, but similar to the rates observed in the entire unmarried group in a prospective cohort of 6051 patients undergoing cardiac catheterization for suspected CHD with a median follow-up of 3.7 years^29^. However, some individuals who were living alone at inclusion may later be living with a relative or a friend, whereas others may lose a spouse through death or divorce and subsequently live alone. Consequently, reasons why people live alone can vary. In an older population, living alone is often the result of widowhood or divorce, whereas for young people it is related to the interim period after they have left their parental home and when they are yet to found a family or partnership of their own^36^. Moreover, living in one’s own apartment in a retirement community is probably different from living alone in a rural area. Including other types of information regarding an individual’s living arrangement and social network would likely improve the predictive value of “living alone” in future studies.

Our study has several limits. The marital status is known only at inclusion and consequently can evolve during the follow-up. We also ignore the evolution of CV factors and their management. It is possible that at least part of the observed effects is due to an unmeasured (and crucial) confounders mainly related to psychological, social and economical issues. For example, divorce can be cause or consequence of psychological distress (anxiety, depression, etc.), social problems (burn-out, cultural differences, unhealthy style of life, social networks, etc.) and economical difficulties (unemployed, housing difficulties, etc.). Marital status should be a marker of these important unmeasured cofactors and explicitly asking about marital status can be difficult in primary care. For example, it might be possible to ask about the degree of social support or whether the subject lives alone instead of asking whether they are married or divorced. This mode of communication is less intrusive. Moreover, because the outcome events in the present analysis did not encompass CV events such as angina pectoris, intermittent claudication and transient ischemic attacks, the observed association between marital status and CV events observed in our analysis must be seen with caution. Also, the cohort, although in an historical and cultural little town of central Italy, was conceptualized more than 40 years. Social norms and lifestyle as well as the quality of healthcare and access to healthcare in this cohort are probably significantly different from contemporary populations. A potential selection bias of the married people in the cohort, either women or men, is possible. Disadvantaged population is rarely represented in studies and married status does not ensure that there are no personal psychological, social or economic problems within the couple. Finally, we were unable to analyse separately different unmarried status (widow, single, and divorced/separate) because of lack of power. With these limitations in mind, being unmarried might be considered a pejorative social marker for CVD prognosis, especially in women. In primary care, marital status should be therefore inquired about as it can be associated with long-term CHD and all-cause incidence and mortality risks in addition to psychological, social and economical issues that futher investigations should definitely assess. That this is crucial in women comes unexpectedly from the present investigation. However, indirect questions such as social support or living alone may be preferable to marital status as they are less intrusive.

## Data Availability

Data can be requested to the CeSEG provided obtaining the authorization by the Italian Authority for Privacy.
